# Effect of Intranasal Instillation of Lipopolysaccharide on Lung Development and Its Related Mechanism in Newborn Mice

**DOI:** 10.1089/jir.2019.0006

**Published:** 2019-10-29

**Authors:** Yaoyao You, Chunbao Guo, Han Zhang, Sijun Deng, Jia Tang, Lingqi Xu, Chun Deng, Fang Gong

**Affiliations:** ^1^ Department of Pediatrics, Yongchuan Hospital of Chongqing Medical University, Chongqing, P.R. China.; ^2^ Department of Neonatology, Children's Hospital of Chongqing Medical University, Ministry of Education Key Laboratory of Child Development and Disorders, Chongqing, P.R. China.; ^3^ Department of Hepatology and Liver Transplantation Center, Children's Hospital, Chongqing Medical University, Chongqing, P.R. China.

**Keywords:** lipopolysaccharide, postnatal pulmonary inflammation, lung development, bronchopulmonary dysplasia

## Abstract

Premature infants are prone to repeated lung infections after birth, which can disrupt the development of lung structure and function. However, the effects of postnatal pulmonary inflammation on lung development in newborn mice have not been reported and may play an important role in the development of bronchopulmonary dysplasia (BPD). This study aimed to establish a BPD model of postnatal pulmonary inflammation in premature infants and to explore its role and possible mechanisms in the pathogenesis of BPD. We exposed postnatal day 1 mice to lipopolysaccharide (LPS) and normal saline for 14 days. Pulmonary inflammation and alveolar microvascular development were assessed by histology. In addition, we also examined the expression of vascular endothelial growth factor (VEGF), VEGFR2, nuclear factor-kappa-B (NF-κB) and related inflammatory mediators [interleukin-1β (IL-1β), tumor necrosis factor-alpha (TNF-α), macrophage inflammatory protein-1α (MIP-1α), monocyte chemoattractant protein-1 (MCP-1)] in the lungs. Lung histology revealed inflammatory cell infiltration, alveolar simplification, and decreased microvascular density in LPS-exposed lungs. VEGF and VEGFR2 expression was decreased in the lungs of LPS-exposed neonatal mice. Furthermore, we detected elevated levels of the inflammatory mediators IL-1β, TNF-α, MIP-1α, and MCP-1 in the lungs, which are associated with the activation of NF-κB. Intranasal instillation of LPS inhibits lung development in newborn mice, and postnatal pulmonary inflammation may participate in the pathogenesis of BPD. The mechanism is related to the inhibition of VEGF and VEGFR2 and the upregulation of inflammatory mediators through activation of NF-κB.

## Introduction

Bronchopulmonary dysplasia (BPD) is a chronic lung disease that most often occurs in premature neonates with persistent oxygen supplementation (Davidson and Berkelhamer 2017). In recent years, with the development of perinatal medicine, the pathological features of BPD have changed. The “new” BPD is characterized by alveolar simplification and abnormal vascular growth (Yun and others 2016). The etiology and pathogenesis of BPD are unknown. A large number of inflammatory factors are detected in the serum of children with BPD, suggesting that BPD development is closely related to the inflammatory response (Hayes and others 2010). The effect of inflammation on BPD has previously concentrated on intrauterine infection, but premature neonates are prone to repeated infection of the lungs due to imperfect immune system development, immature lung development, and long-term tracheal intubation or mechanical ventilation after birth (Shahzad and others 2016). Therefore, postnatal pulmonary inflammation may also be an important risk factor for BPD. To further investigate the possible mechanisms of BPD in postnatal lung inflammation in preterm neonates, we designed and conducted this animal experiment.

Physiological angiogenesis is essential for postnatal lung development, and inhibition of pulmonary angiogenesis impairs alveolarization, leading to BPD (Stenmark and Abman 2005). Vascular endothelial growth factor (VEGF), a key factor in angiogenesis, is expressed in lung epithelial and mesenchymal cells, and its receptor VEGFR2 is expressed in endothelial cells. VEGF and VEGFR2 combine to drive postnatal angiogenesis and alveolarization. VEGF and VEGFR2 expression levels are decreased in the lungs of infants dying from BPD (Bhatt and others 2001). Inhibiting VEGF in neonatal rats causes sparse pulmonary vasculature and impairs alveolar formation, while enhancement of VEGF signaling can rescue hyperoxia-induced alveolar destruction (Kroon and others 2015). VEGF signaling plays an important role in normal pulmonary vascular development.

Angiogenesis is also closely related to inflammation (Rudloff and others 2017). In neonatal mice with chorioamnionitis, inflammatory mediators in the lungs disrupt the development of pulmonary vessels and alveoli (Miller and others 2010). Studies have reported abnormal alveolar capillaries in patients who died of BPD (Coalson 2000; De Paepe and others 2008). How postnatal pulmonary inflammation affects the developing alveoli and lung vasculature has not yet been reported.

The nuclear factor-kappa-B (NF-κB) signaling pathway is a key regulator of inflammation. Studies have shown that NF-κB and downstream inflammatory factors are associated with pathological angiogenesis (Alvira 2014). NF-κB normally remains inactive in its binding to the inhibitory protein IκB in the cytoplasm. The activity of NF-κB is mainly regulated by the IκB family of inhibitory proteins, and its typical member is IκBα. IκBα is phosphorylated at the Ser32 site and degrades, which activates NF-κB (Wright and others 2009). P65 is a subunit of NF-κB, and phosphorylation of P65 at the Ser536 site suggests an increase in NF-κB activity (Lin and others 2013). Increased NF-κB activity plays a key role in BPD (Bourbia and others 2006). However, although many studies have shown that increased NF-κB activity is associated with pathological angiogenesis in the lungs, the specific mechanism has not been previously described.

In this study, we found that chronic exposure to lipopolysaccharide (LPS) at 25 mg/kg can cause pathological changes similar to those in BPD. Postnatal pulmonary inflammation impedes the development of alveolar and pulmonary microvasculature, and its mechanism may be related to the inhibition of VEGF signaling and the activation of NF-κB to upregulate proinflammatory cytokines and chemokines, which may be an important risk factor for BPD. Our findings provide a new perspective on the vascular hypothesis of BPD and identify new targets for BPD research.

## Materials and Methods

### Animals and treatment

All animal studies were conducted in accordance with standards approved by the Chongqing Medical University Animal Use Committee. Full-term C57BL/6J mice were obtained within 24 h of birth from the experimental animal center of Chongqing Medical University. The pups were continuously instilled with 25 mg/kg LPS (Sigma) intratracheally on postnatal days (P) 1–14. The remaining pups were given an equivalent volume of normal saline. The mice were weighed at P1, P3, P7, and P14.

### Tissue collection and histology

Mice were sacrificed, and lung tissues were collected for analysis on P14. The left lobe was frozen at −80°C for quantitative real-time polymerase chain reaction (qRT-PCR), Western blotting, and ELISA. The right lobe was fixed with 4% paraformaldehyde for Hematoxylin and Eosin staining to observe the morphological structure of the lung tissue. All fixed lung tissue samples were embedded in paraffin and sectioned for histochemical analysis by light microscopy. To compare specific structural features of the lung, we assessed alveolarization using radioactive alveolar counts (RACs) and mean linear intercepts (MLIs) (Balasubramaniam and others 2007). The MLIs were calculated as the linear sum of the lengths of all lines randomly drawn on the image, divided by the number of intersections between the alveolar walls and the lines. From the center of the respiratory bronchi, when a vertical line is dropped to the edge of the acinus connective tissues or septum or pleura, the number of intervals intersected by this line was calculated as the RAC. At least 5 measurements were taken in each animal.

### Immunohistochemical staining

Immunohistochemistry was performed on formalin-fixed lung sections using techniques previously described (Yun and others 2016). Briefly, the slides were placed at 4°C overnight with primary antibodies against the following: PECAM-1/CD31 (1:50; Abcam, UK), VEGF (1:300; Proteintech Group), tumor necrosis factor-alpha (TNF-α, 1:200; Wanleibio, China), and interleukin-1β (IL-1β, 1:200; Wanleibio). After washing with phosphate-buffered saline (PBS), the sections were incubated with diluted secondary antibody. Next, the sections were visualized with 3,39-diaminobenzidine (Life Technologies, Carlsbad, CA) and then counterstained with Hematoxylin. Finally, the slides were analyzed on a Nikon 55I microscope with a DS-Fi1c camera and NIS-Elements F software. Image-Pro Plus software (version 6.0; Media Cybernetics, Rockville, MD) was used to detect the integrated optical density of positive tissues. According to the method described previously (Maniscalco and others 2002), microvessel density (MVD) was determined under high magnification.

### Western blot analyses

Lung tissues were minced and lysed in lysis buffer. The protein concentrations in the supernatants were detected using the BCA Protein Assay Kit (Thermo Scientific, Rockford, IL). Next, equivalent amounts of proteins from each sample were separated using sodium dodecyl sulfate/polyacrylamide gel electrophoresis and transferred to polyvinylidene difluoride membranes (Millipore). After blocking with 5% bovine serum albumin (Sigma) for 1 h, the membranes were incubated with their respective primary antibodies, including antibodies against phospho-P65 (Ser536) (1:300; Abcam, UK), P65 (1:500; Proteintech Group), phospho-IκBα (Ser32) (1:500; Cell Signaling Technology), IκBα (1:500; Proteintech Group), and VEGFR2 (1:500; Proteintech Group), overnight at 4°C. The membranes were then incubated with a secondary antibody at room temperature for 1 h. Blots were visualized using the ECL Western Blotting Kit (Millipore), and the results were subjected to gray value analysis using Quantity One analysis software.

### Quantitative real-time PCR

Lung samples were used to extract total RNA using TRIzol reagent (Invitrogen), and cDNA was generated by reverse transcription according to the protocol of the commercial Reverse Transcription Reagent Kit (TaKaRa, Japan). qRT-PCR was performed using SYBR Premix Ex Taq™ (Takara, Japan) according to the manufacturer's instructions in a CFX96 Touch Real-Time PCR Detection System (Bio-Rad). The expression level of each gene was normalized to that of the housekeeping gene of GADPH using the relative quantification (2^− ΔΔCt^) method (Qiu and others 2011). The primers were synthesized by Beijing Huada Gene, and the sequences are shown in Table 1.

**Table 1.** Mouse Primer Sequences for Quantitative Real-Time Polymerase Chain Reaction

*Gene*	*Forward primer (5′-3′)*	*Reverse primer (5′-3′)*
VEGF	ACATCTTCAAGCCGTCCTGT	AGGTTTGATCCGCATGATCT
VEGFR2	CTTGCAGGGGACAGCGGGAC	AATCGACCCTCGGCAGGGGA
MIP-1α	AGATTCCACGCCAATTCATC	CCCAGGTCTCTTTGGAGTCA
MCP-1	CCCAATGAGTAGGCTGGAGA	TCTGGACCCATTCCTTCTTGv
IL-1β	GGCTGGACTGTTTCTAATGC	AGCTTCTCCACAGCCACAAT
TNF-α	ACGGCATGGATCTCAAAGAC	GTGGGTGAGGAGCACGTAGT
GADPH	CAGCGACACCCACTCCTCCACCTT	CATGAGGTCCACCACCCTGTTGCT

### ELISA analysis of lung homogenates

The lung tissues were washed with prechilled PBS (pH = 7.4) and homogenized with PBS buffer in a tissue homogenizer. Homogenates were centrifuged at 4,000 rpm for 10 min at 4°C, and supernatants were collected. These assays measured lung homogenate using the Mouse JE/MCP-1 ELISA Kit (NeoBioscience Technology, China) and Mouse MIP-1α (CCL3) ELISA Kit (NeoBioscience Technology) according to the manufacturer's instructions.

### Statistical analyses

Statistical analyses were performed using the GraphPad Prism version 6.00 (GraphPad Software, San Diego, CA). All data are expressed as the mean ± SEM. Statistical significance between 2 groups was analyzed by Student's *t*-test. Repeated-measures analysis of variance was performed to analyze the difference in body weight on P1, P3, P7, P14. *P* values were considered significant if they were less than 0.05.

## Results

### Body weights of newborn mice exposed to LPS were reduced

**FIG. 1.**
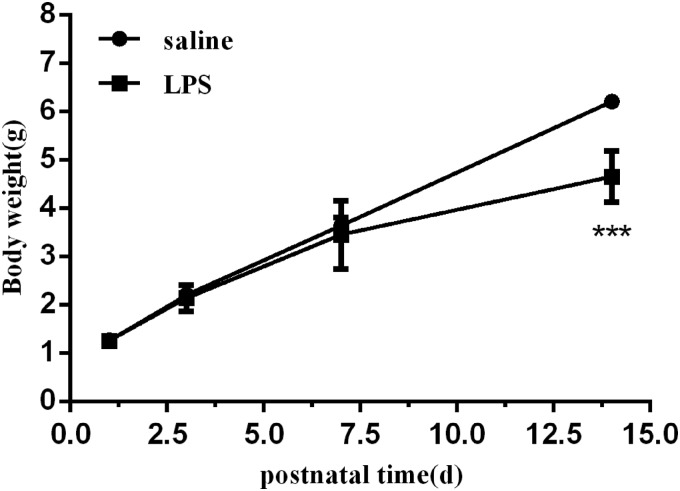
The body weights of mice exposed to LPS and saline. The mice were weighed at P1, P3, P7, and P14. Values represent the mean ± SEM (*n* = 8 per group). ***P < 0.001. LPS, lipopolysaccharide; P, postnatal days.

To determine the effect of postnatal LPS on body weight, we monitored the weights of neonatal mice. There was no difference in body weight between the LPS group and the saline group at birth or P3. Pups exposed to LPS showed a decrease in body weight compared with the saline group starting from P7, but the difference was not statistically significant. However, we found a significant difference in body weights between the 2 groups when the mice were 14 days old (Fig. 1).

### Lung development of newborn mice exposed to LPS was impaired

**FIG. 2.**
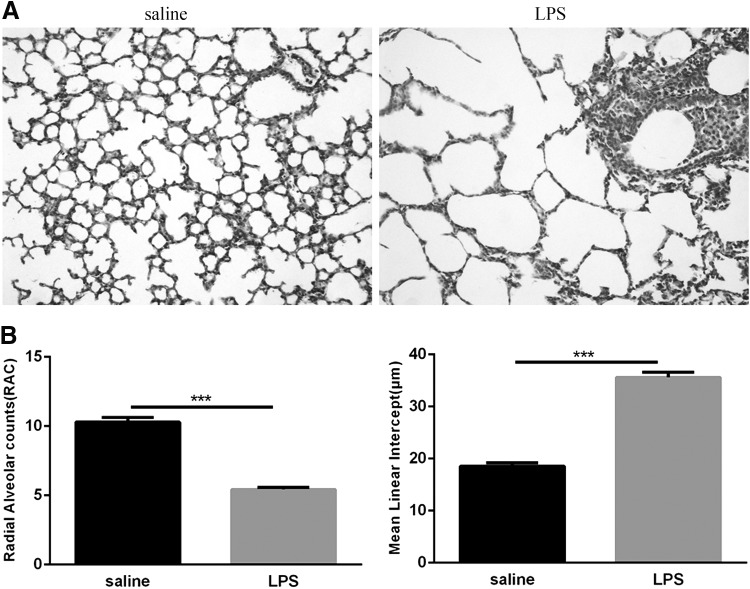
Histological measurements of the neonatal lungs following LPS and saline exposure. **(A)** Histology sections of neonatal lungs were subjected to Hematoxylin and Eosin staining for morphometric analyses. Magnification × 200. **(B)** RAC and MLI assays. Values represent the mean ± SEM (*n* = 8 per group). ***P < 0.001. MLI, mean linear intercept; RAC, radioactive alveolar counts;

Because postnatal LPS exposure can reduce the weight of newborn mice, to further determine whether it could damage alveolar development, we evaluated the lung histology of newborn mice exposed to LPS for 14 days. The histological characteristics of the lungs show a simplification of the alveoli, characterized by a decrease in the number of alveoli, an enlargement of the alveolar space, and significant perivascular inflammatory cell infiltration. In contrast, saline-exposed control mice had essentially normal lung structures with no or only mild perivascular inflammatory cell infiltration (Fig. 2A). Morphometric analyses revealed a significant decrease in RAC and prominently increased MLI in the LPS group compared with those measures in the saline group (Fig. 2B). These results indicate that postpartum intranasal instilled LPS-induced pulmonary inflammation inhibits alveolar development.

### Lung MVD in LPS-exposed mouse lungs was reduced

**FIG. 3.**
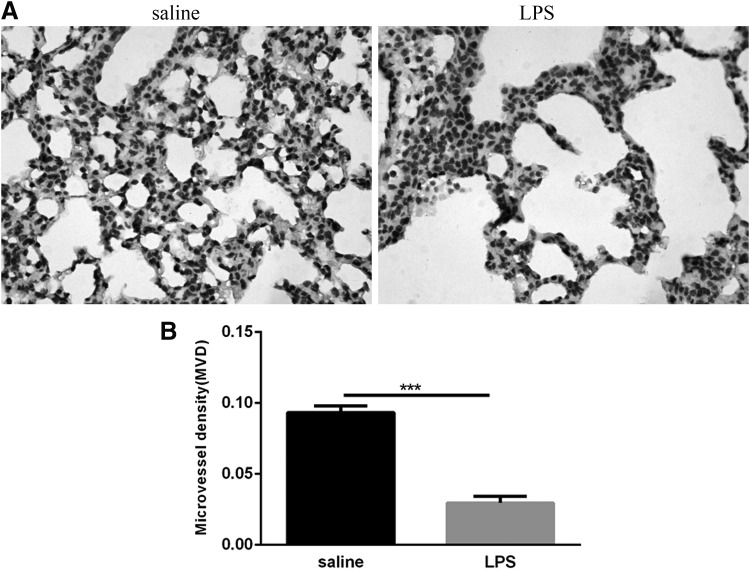
Pulmonary microvascular measurements of the neonatal lungs following LPS and saline exposure. **(A)** CD31 expression in the lungs was determined by immunohistology. Magnification × 400. **(B)** MVD assay. Values represent the mean ± SEM (*n* = 8 per group). ***P < 0.001. MVD, microvessel density.

CD31 is one of the earliest markers for detecting endothelial cells in the fetus and therefore serves as a marker for vascular development (Baldwin and others 1994). To initially observe the effects of postnatal LPS-induced pulmonary inflammation on microvascular development, we detected the expression of CD31 in lung tissue by immunohistochemistry. CD31 was detected in histologically identified endothelial cells in lung tissue (Fig. 3A), and we used MVD as an indicator of vascular development. The MVD value of the LPS group was lower than that of the saline group (Fig. 3B). These findings indicate that postnatal LPS-induced pulmonary inflammation inhibits pulmonary microvascular development.

### Expression of VEGF and VEGFR2 in mice exposed to LPS was decreased

**FIG. 4.**
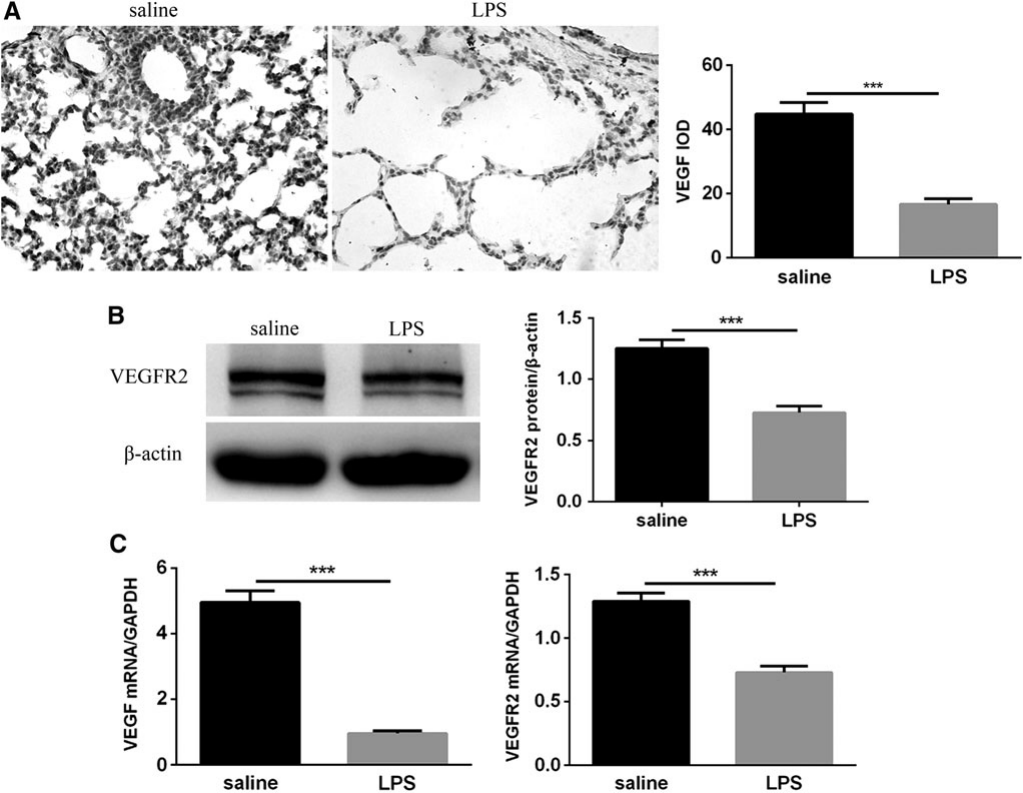
Expression of VEGF and VEGFR2 in neonatal lungs following LPS and saline exposure. **(A)** Immunohistochemical analysis of VEGF in the lungs and their IOD values. Magnification × 400. **(B)** VEGFR2 protein levels in the lungs were measured by Western blotting. β-actin was used as a control. **(C)** VEGF and VEGFR2 mRNA levels were assessed by qRT-PCR analysis. Values represent the mean ± SEM (*n* = 8 per group). ***P < 0.001. IOD, integrated optical density; qRT-PCR, quantitative real-time polymerase chain reaction; VEGF, vascular endothelial growth factor.

To determine if postnatal LPS decreases VEGF/VEGFR2 signaling in the lungs of neonatal mice, animals from the saline and LPS groups were sacrificed at 14 days to obtain lung tissue specimens for the detection of VEGF and VEGFR2 expression in the lungs. We first measured the level of VEGF protein by immunohistochemistry. As shown, lung VEGF was predominantly expressed in the airway epithelium, distal lung epithelium and vascular endothelial cells after exposure to saline and was significantly decreased after exposure to LPS (Fig. 4A). The expression of VEGFR2 protein was analyzed by Western blot. As shown, neonatal exposure to LPS downregulated lung VEGFR2 expression, as evidenced by a decrease in lung VEGFR2 protein content in the LPS group versus the saline group (Fig. 4B). qRT-PCR analysis showed that VEGF and VEGFR2 mRNA were significantly decreased in the lungs of LPS-exposed pups compared with the saline-exposed mice (Fig. 4C). These results indicated that postnatal LPS-induced pulmonary inflammation interferes with lung VEGF/VEGFR2 signaling.

### NF-κB activity in the lungs of newborn mice exposed to LPS was increased

**FIG. 5.**
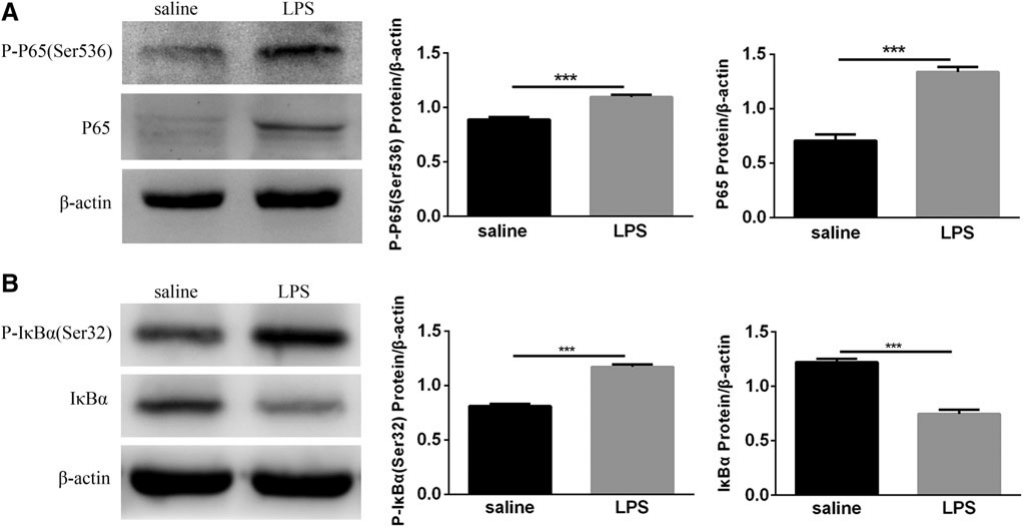
Detection of NF-κB complexes in the lungs of neonatal mice. **(A)** The expression of P-P65 (Ser536) and P65 protein in the lungs. **(B)** The expression of P-IκBα (Ser32) and IκBα protein in the lungs. Western blotting was performed to detect the protein expression of P-P65 (Ser536), P65, P-IκBα (Ser32), and IκBα. β-actin was used as a control. Values represent the mean ± SEM (*n* = 8 per group). ***P < 0.001. NF-κB, nuclear factor-kappa-B.

To determine whether postnatal LPS induces NF-κB activation in the lung, neonatal mice were treated with LPS for 14 days. The levels of P65 and P-P65 (Ser536) in the lungs were significantly increased after LPS exposure (Fig. 5A). Meanwhile, Western blot results showed a significant increase in P-IκBα (Ser32) after LPS exposure, while IκBα was statistically reduced (Fig. 5B). These results suggested that postnatal LPS activates the NF-κB signaling pathway. When the signaling pathway was activated, the IκBα protein was degraded, and the NF-κB dimer entered the nucleus to regulate target gene expression.

### Expression of the proinflammatory cytokines, TNF-α and IL-1β, in mice exposed to LPS was increased

**FIG. 6.**
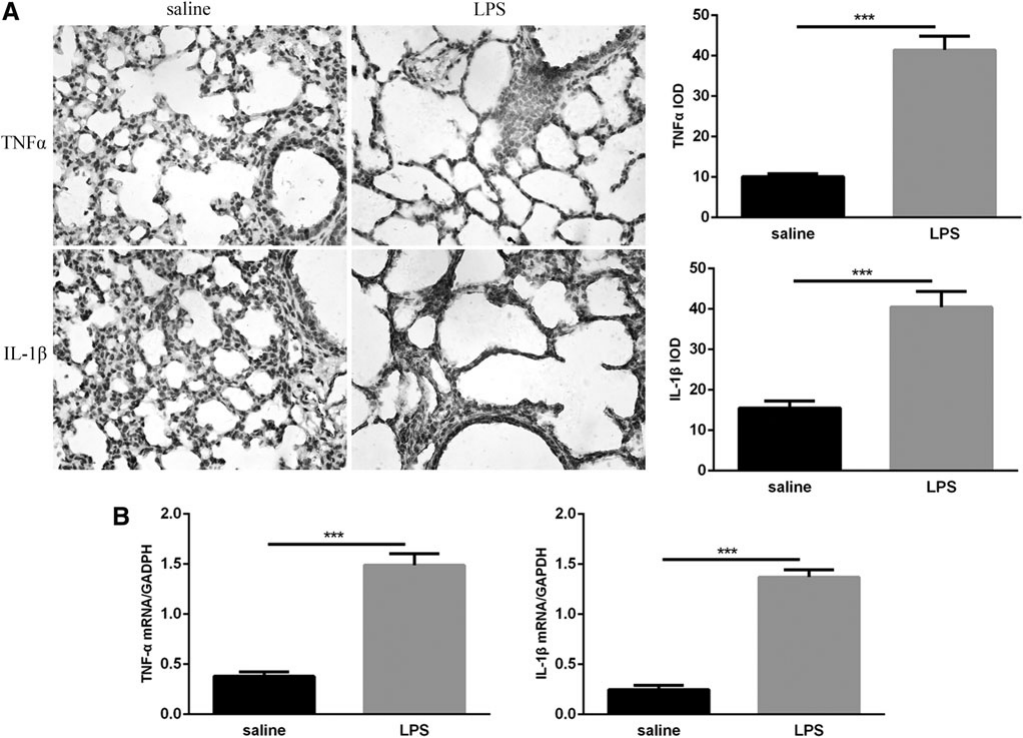
Expression of TNF-α and IL-1β in neonatal lungs following LPS and saline exposure. **(A)** Immunohistochemical analysis of TNF-α and IL-1β in the lungs and their IOD values. Magnification × 400. **(B)** TNF-α and IL-1β mRNA levels were detected by qRT-PCR analysis. Values represent the mean ± SEM (*n* = 8 per group). ***P < 0.001. IL-1β, interleukin-1β; TNF-α, tumor necrosis factor-alpha.

Because inflammation is a hallmark of BPD, we used immunohistochemistry to detect the expression of cytokines, TNF-α and IL-1β, in neonatal lungs at day 14 to study the effects of postnatal LPS on pulmonary inflammatory markers. TNF-α and IL-1β proteins are expressed primarily in alveolar epithelial cells, vascular endothelial cells, bronchial epithelial cells, and activated inflammatory cells, and only faint staining was observed in the lung tissues of mice exposed to saline, whereas strong positive staining was observed in the lungs of LPS-exposed mice (Fig. 6A). qRT-PCR analysis showed that TNF-α and IL-1β mRNA were significantly increased in the lungs of LPS-exposed pups compared with saline-exposed mice (Fig. 6B). These findings suggest that postnatal LPS-induced pulmonary inflammation can increase the expression of the proinflammatory cytokines IL-1β and TNF-α.

### Expression of the chemokines, MIP-1α and MCP-1, in mice exposed to LPS was increased

**FIG. 7.**
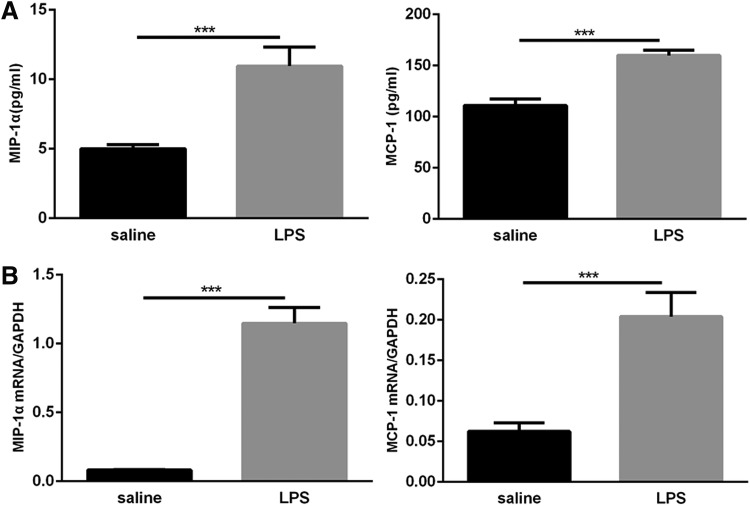
Expression of MIP-1α and MCP-1 in neonatal lungs following LPS and saline exposure. **(A)** MIP-1 α and MCP-1 levels in total lung homogenate were measured by ELISA. **(B)** MIP-1α and MCP-1 mRNA levels were detected by qRT-PCR analysis. Values represent the mean ± SEM (*n* = 8 per group). ***P < 0.001.

Macrophage inflammatory protein-1α (MIP-1α) and monocyte chemoattractant protein-1 (MCP-1) are CC chemokines with pleiotropic activities and have chemotaxis and activation effects on various inflammatory cells and monocytes/macrophages. They are closely related to BPD pulmonary microvascular development (Miller and others 2010). We first measured the concentrations of MIP-1α and MCP-1 in lung homogenates obtained from neonatal mice after intranasal instillation of LPS or saline for 14 days. LPS exposure elicited a strong increase in MIP-1α and MCP-1 levels in lung homogenates compared with saline exposure (Fig. 7A). qRT-PCR analysis showed that MIP-1α and MCP-1 mRNA levels were significantly increased in the lungs of LPS-exposed pups compared with saline-exposed mice (Fig. 7B). These consequences suggest that postnatal LPS-induced pulmonary inflammation can increase the expression of the chemokines MIP-1α and MCP-1.

## Discussion

Lung development includes the development of alveolar and pulmonary microvasculature. The saccular and alveolar phases of lung development are important stages in the formation of pulmonary vascular structures and secondary alveolar septa (Madurga and others 2013; Greer and others 2014). Therefore, we speculate that these stages are critical for the growth and development of alveolar and pulmonary microvasculature. Neonatal mice whose lungs develop in the saccular stage after birth and enter the alveolar stage 5 days after birth can be used as a natural preterm model (Bhandari 2014). Continuous high-dose LPS intervention in neonatal mice during this period inhibited lung development. Research of LPS on lung injury in neonatal mice has been focused on acute lung injury. A classic model of acute lung injury was established by intraperitoneal injection of 5 mg/kg LPS in neonatal mice at 7 days of age. The pathological manifestations were pulmonary inflammatory cell infiltration, thickening of the lung septum, pulmonary edema, and other signs of pulmonary inflammation (Cai and others 2005).

In contrast to this model, our study found that continuous nasal infusion of 25 mg/kg LPS for 13 days in neonatal mice not only induced lung inflammation but also significantly inhibited alveolar development. This phenomenon has not been previously reported. Duration of LPS exposure may also be a key factor in modeling, consistent with this study, and hyperoxia exposure lasting for 2 weeks produces an animal model of BPD (Han and others 2015). In the present study, neonatal mice were intranasally instilled with LPS for 2 weeks, and pathological changes similar to those in BPD appeared in the lungs, suggesting that postnatal pulmonary inflammation may be an important risk factor for BPD development. There is a close relationship between inflammation and vascular development. Abnormal microvasculature development may be an important cause of BPD in postnatal lung inflammation.

Abnormal blood vessels are observed in patients with BPD, including pulmonary vascular stagnation and deformed capillaries. Coalson and others (1999) found that capillary reduction and malformation in patients who died due to BPD were similar to our findings. Newborn mice exposed to hyperoxia had decreased lung capillary surface area (Pappas and others 1983).We found that in the lung tissue of newborn mice exposed to LPS, the intensity of CD31 immunostaining was reduced, and the MVD in the lungs was significantly reduced. This result suggests that postnatal lung inflammation reduces the number of vascular endothelial cells and leads to pulmonary angiogenesis disorders.

Pulmonary vasculature is formed by blood vessels that are tightly connected to the alveolar epithelium, and the interaction between the alveolar epithelium and the vascular endothelium promotes the formation of alveoli (Roth-Kleiner and Post 2005). A potential mechanism of alveolar capillary decline in this study is the decreased expression of angiogenic growth factors and their receptors. After inhibiting the expression of VEGF or VEGFR2 in neonatal mice, the lungs showed obvious alveolar simplification and vascular development retardation (Thebaud and others 2005; Kroon and others 2015). This result highlights the important role of VEGF and VEGFR2 in postnatal angiogenesis. We found that the protein and mRNA expression of VEGF and its receptor VEGFR2 were decreased in the lungs after exposure to LPS in neonatal mice. Consistent with this study, VEGF was reduced in neonatal mice after hyperoxia (Hou and others 2008). Studies have found that VEGF inhibits TNF-α-induced apoptosis in endothelial cells (Spyridopoulos and others 1997), and the decrease in VEGF and VEGFR2 has implications for endothelial cell proliferation and migration (Bhatt and others 2001; Shimotake and others 2010).

In this study, in addition to the decrease in VEGF and VEGFR2, various inflammatory mediators (IL-1β, TNF-α, MIP-1α, and MCP-1) were increased, and the mechanism may be that the reduction in VEGF and VEGFR2 in the lungs results in endothelial cell death or apoptosis induced by inflammatory agents. These data suggest that postnatal pulmonary inflammation may affect the survival of endothelial cells by inhibiting VEGF/VEGFR2 signaling to block the physiological formation of blood vessels, thereby inhibiting the development of pulmonary microvasculature.

It has been reported that LPS binds to Toll-like receptor 4 to activate NF-κB through MyD88-dependent pathway, inducing secretion of inflammatory and chemokines. At the same time, it can also activate IRF3 through MyD88-independent pathway (TRIF pathway) to induce the production of type I interferons (IFNs) (Kawai and others 2001; Doyle and others 2002; Vaure and Liu 2014). Among them, NF-κB is an important signaling molecule that regulates inflammation and angiogenesis. Studies have found that NF-κB activation is associated with the formation of pathological vessels in neurostromal tumors (Bonavia and others 2012). Wright and others (2010) found that nitric oxide can improve lung microvascular endothelial cell injury induced by hyperoxia in neonatal mice by inhibiting NF-κB activity. In the present experiment, LPS activated the NF-κB pathway in the lungs.

Translocation of activated NF-κB subunits to the nucleus induces the expression of inflammatory mediators, IL-1β, TNF-α, MIP-1α, and MCP-1, initiating a proinflammatory cascade and ultimately leading to pathological tissue damage in the lungs. Furthermore, stimulation of the lungs of newborn mice with IL-1β could cause abnormal microvascular development (Bry and Lappalainen 2007). TNF-α affects angiogenesis in the lungs by damaging vascular endothelial cells (Su and others 2015). Injecting LPS into pregnant mice during the embryonic period stimulates the formation of pathological blood vessels in the lungs by MIP-1α and MCP-1 (Miller and others 2010). This angiogenesis is different from that of normal blood vessels. It is far away from the alveolar epithelium, which increases the distance between the airway and the blood vessels, and reduces the interaction between epithelial cells and endothelial cells (Roth-Kleiner and Post 2005). Such dysmorphic capillaries are commonly observed in the lungs of patients with BPD. In this study, we found that the distance between the blood vessels and the alveolar epithelium increased after exposure to LPS, suggesting the formation of pathological microvessels.

The above studies indicate that inflammation-mediated angiogenesis in the lungs of neonatal mice could disrupt the normal relationships between the developing airways and capillaries and eventually develop into BPD. In summary, postnatal pulmonary inflammation may be caused by the upregulation of inflammatory mediators through the activation of NF-κB, leading to the formation of abnormal microvessels, thereby disrupting normal vascular development.

This report is the first to demonstrate that continuous intranasal instillation of LPS after birth can lead to disrupted lung development in neonatal mice, and postnatal pulmonary inflammation could play a prominent role in the development of BPD. This study further emphasizes the important role of inflammation in BPD. In addition to the previously reported chorioamnionitis, postnatal pulmonary inflammation also significantly inhibits alveolar and microvascular development. Its mechanism may be related to the decreased expression of VEGF and VEGFR2 and the upregulation of inflammatory mediators by NF-κB activation. VEGF, VEGFR2, and NF-κB may become new targets for the study and treatment of BPD. There are still many limitations of this study.

The specific mechanism of pulmonary inflammation inhibiting lung development after birth is still unclear. In addition to activating the NF-κB pathway, LPS also activates IFR3 to induce the production of type I IFNs. Studies have found that it has anti-tumor angiogenesis (Dvorak and Gresser 1989; Indraccolo 2010). It may play a role in the development of BPD pulmonary microvasculature and can be further verified in future experiments.
